# The influence of Zircos‐E® etchant, silica coating, and alumina air‐particle abrasion on the debonding resistance of endocrowns with three different preparation designs

**DOI:** 10.1002/cre2.901

**Published:** 2024-05-21

**Authors:** Ahmed Yaseen Alqutaibi, Mohammed Ahmed Alghauli, Karim Dewedar, Mohammed H. AbdElaziz, Samah Saker

**Affiliations:** ^1^ Substitutive Dental Science Department, College of Dentistry Taibah University Al‐Madinah Saudi Arabia; ^2^ Department of Prosthodontics, Faculty of Dentistry Ibb University Ibb Yemen; ^3^ Crown and Bridge Department, Faculty of Dental Medicine Al‐Azhar University Cairo Egypt; ^4^ Fixed Prosthodontic Department, Faculty of Dentistry Mansoura University Mansoura Egypt

**Keywords:** airborne‐particle abrasion, debonding resistance, Zirconia endocrowns, Zircos‐E® etch

## Abstract

**Objectives:**

The study aimed to evaluate the debonding resistance of three different endocrown designs on molar teeth, using three different zirconia surface pretreatments.

**Material and Method:**

Ninety human mandibular first molars were divided into three main groups: endocrowns without ferrule, with 1 mm ferrule, and with 2 mm ferrule. The subgroups were defined by their surface pretreatment method used (*n* = 15): 50 μm alumina air‐particle abrasion, silica coating using 30 μm Cojet™ particles, and Zircos‐E® etching. The endocrowns were fabricated using multilayer zirconia ceramic, cemented with self‐adhesive resin cement, and subjected to 5000 thermocycles (5–55°C) before debonding. The data obtained were analyzed using a two‐way ANOVA.

**Results:**

All test specimens survived the thermocyclic aging. The results indicated that both the preparation design and the surface treatment had a significant impact on the resistance to debonding of the endocrowns (*p* < .001). The 2 mm ferrule followed by the 1 mm ferrule designs exhibited the highest debonding resistance, both were superior to the endocrown without ferrule. Zircos‐E® etching and silica coating yielded comparable debonding resistance, which were significantly higher than alumina air‐particle abrasion. All endocrowns demonstrated a favorable failure mode.

**Conclusions:**

All designs and surface treatments showed high debonding resistance for a single restoration. However, ferrule designs with Zircos‐E® etching or silica coating may represent better clinical options compared to the nonferrule design or alumina airborne‐particle abrasion. Nonetheless, further research, including fatigue testing and evaluations with different luting agents is recommended.

## 1 INTRODUCTION

The purpose of saving ETT is to keep the tooth in function for a reasonable amount of time. (Ferrari et al., 2022) Over the past few decades, a shift in perspective has emerged regarding the treatment of endodontically treated teeth (ETT). Initially, the traditional post‐and‐core crown approach was standard practice; however, it has come to be seen as unnecessary and, in fact, destructive. Significantly, research has indicated that the presence of two or more short axial walls may render the need for a post obsolete. (Mangold & Kern, 2011) Transitioning to recent developments has been discovered through evidence‐based studies that partial coverage restorations boast a favorable clinical prognosis for midterm service. Moreover, these findings suggest that long‐term clinical success is anticipated when employing adhesive techniques alongside restorative materials that possess robust mechanical properties. (Mario et al., 2022) Innovatively, endocrowns have risen as a novel solution, utilizing on the wide pulp chamber of teeth to augment the surface area available for adhesion. This concept, which revolutionizes the approach to dental restoration, was introduced in a practical framework by Pissis in 1995. (Pissis, 1995) His pioneering work focused on rehabilitating severely damaged upper incisors. Notably, the early use of metal post‐and‐core in these restorations presented an esthetic drawback by obstructing light transmission. This challenge was ingeniously addressed by incorporating glass‐ceramic materials into the restoration, extending into the pulp cavity of the incisors, thus enhancing the esthetic outcome. Finally, this technique underwent further refinement to adapt it for posterior teeth, a modification credited to Bindl and Mörmann in 1998 (Bindl & Mörmann, 1999). A successive study in 2005 included both molar and premolar teeth and found it was more suitable for molar teeth due to their wide pulp chamber and the reasonable surface area for adhesion (Bindl et al., 2005). Furthermore Otto and Mörmann 2015 performed long‐term clinical evaluation lasting up to 10 years, which confirmed the clinical applicability of endocrowns (Otto & Mörmann, 2015).

The reliability and durability of resin‐luting composite adhesion to the newly introduced ceramic materials over the last decade have rendered endocrowns one of the primary options for rehabilitating ETT (Mannocci et al., 2021; Soliman et al., 2021). In the pursuit of perfection and improved long‐term survival of ETT, researchers have extended the endocrown inlay into the pulp chamber with various depths to increase the surface area. These extensions, particularly in mandibular premolars, have made the designs resemble a short post rather than the conservative approach intended by the concept of endocrowns. Furthermore, the pulpal inlays at different depths have shown no beneficial impact on the fracture resistance of molar teeth, (Hayes et al., 2017) and have adversely affected the scanning accuracy, hence the digital workflow and the accuracy of final restorations (Gurpinar & Tak, 2022). Extending the endocrown preparations to the external axial surface by engaging a circumferential ferrule with different heights has been the most reasonable design to follow; laboratory studies have demonstrated an improvement in the fracture resistance of ETT restored with endocrowns with ferrules (Einhorn et al., 2019). Moreover, the use of a 1 mm ferrule design has reported fewer catastrophic failure incidences compared to the 2 mm ferrule design (Einhorn et al., 2019).

Many laboratory studies have recommended zirconia as the material of choice for endocrown restoration due to its unique bioinert physical structure and excellent mechanical properties (Ahmed et al., 2022; Alqutaibi et al., 2022; Dartora et al., 2021; Lin et al., 2020). In terms of fracture resistance, zirconia endocrowns have shown comparable results in both ferrule and non‐ferrule designs, thanks to the appreciable physical‐mechanical properties of monolithic zirconia, and the reliable and durable bond between zirconia and tooth substrates, especially when using luting resins containing 10‐MDP phosphate monomer (Ahmed et al., 2022). However, it should be noted that zirconia specimens with high fracture resistance often come with a higher rate of catastrophic unfavorable failure modes (Ahmed et al., 2022; Dartora et al., 2021). To address this issue in single restorations like endocrowns, it is recommended to use zirconia materials with higher yttria contents, such as 4YSZ or 5YSZ. These materials possess lower mechanical properties than 3Y‐TZP but higher than glass ceramics. Resulting in enhanced material translucency and optic properties, while reducing the incidences of catastrophic failure in endocrowns (Haralur et al., 2020).

Apart from zirconia's good mechanical properties, its inert surface requires modification for optimal adhesion to composite luting resins and their corresponding primers. The gold standard for zirconia surface treatment involves air‐borne particle abrasion using different sizes of aluminum oxide particles and air pressure. While the sizes of alumina particles and jet pressure do not significantly affect the bond strength and durability of zirconia (Shimoe et al., 2019), it is advisable to combine low‐pressure air‐particle surface abrasion with primers and luting resins containing 10‐MDP for bonding. On the other hand, self‐adhesive resin cement may require higher jet pressure, such as 0.25 MPa (Yang et al., 2010). Zirconia surface treatment with tribochemical silica coating might exhibit better morphological features (Queiroz et al., 2015) and bond strength (Bielen et al., 2015) than the AL_2_O_3_ protocol. However, other studies reported comparable bond strength between the two protocols (Thammajaruk et al., 2020), in particular when bonding zirconia surfaces to Panavia F or self‐adhesive resin cement (Kumbuloglu et al., 2006). Furthermore, there are conflicting reports on the etching of zirconia surfaces with the Zircos‐E® Etching solution. Some studies have reported a significant improvement (Sales et al., 2022), while others found no significant difference in composite resin bond strength to zirconia compared to the AL_2_O_3_ treatment protocol (Sadid‐Zadeh et al., 2021).

The strength and durability of the bond between composite luting resin and zirconia may be influenced by various factors, such as micro‐mechanical interlocking (Shimoe et al., 2019), the applied adhesives and chemical adhesion (Yang et al., 2018), and surface features and characteristics (da Silva et al., 2021). This in‐vitro study aimed to assess the debonding resistance of monolithic zirconia endocrowns with different designs and surface treatments after artificial aging. The null hypotheses were as follows: (a) There will be no differences in debonding resistance among the different endocrown designs. (b) There will be no differences in debonding resistance among the different surface treatments. (c) The different surface treatments and preparation designs will exhibit the same patterns of failure.

## 2 MATERIALS AND METHODS

### 2.1 Teeth selection, preparation, and grouping

The study was approved by the Taibah University College of Dentistry Research Ethics Committee (approval number 21032 for the year 2022). The collected teeth were subjected to ultrasonic cleaning and disinfection, then stored in distilled water at a temperature of 4–5°C until used in the experiment, the teeth were used only within 6 months after extraction. A total of 90 mandibular molar teeth with similar anatomy and dimensions were selected for the experiment. The teeth were examined for any pre‐existing fractures or cracks. The recruited teeth had dimensions measuring 10.47 ± 0.9 mm buccolingually and 11.13 ± 1 mm mesiodistally.

The crowns of the teeth were horizontally cut using a low‐speed 0.5 mm diamond disc on a precision saw machine (IsoMet 1000 Buehler). The cutting level was set at 3 mm above the cemento‐enamel junction (CEJ) of the mesial surface. The endodontic treatment of the teeth was done by the same operator using the Protaper system (Dentsply‐Maillefer) in a standardized sequence. Sodium hypochlorite irrigation solution was used during the treatment. Throughout the endodontic procedure, the specimens were held in the operator's hand with wet gauze, put in saline solution upon instruments exchange or short pause of the work during the same procedure, and finally stored in a saline solution at 4–5°C before the next step to prevent dehydration. After the root canal treatment, excess sealer and debris were removed from the access cavity walls with ethyl alcohol. The teeth were then mounted in auto‐polymerizing acrylic resin (Ivocron; Ivoclar Vivadent AG) and positioned parallel to their long axis 3 mm apical to the cementoenamel junction using a paralleometer (PARASKOP M. 100‐120, BEGO) to ensure centralization and alignment with the mold.

The root canal orifices and pulpal floor were sealed with a dual‐cured composite core material following the manufacturer's instructions (Clearfil DC Core Plus). Before applying the core material, the tooth surface was treated with a two‐step, self‐etch adhesive (Clearfil SE; Kuraray America). The applied core material was shaped to create a flat restoration surface at the pulpal floor that was parallel to the occlusal table of the endocrowns. The materials used in the study are listed in Table 1.

**Table 1:** The materials used in the experiment, their composition, and productive company.

Trade name	Scientific name	Composition	Productive company
Zolid FX	5YSZ: zirconium oxide ceramic	ZrO_2_ + HFO_2_ + Y_2_O_3_ ≥ 99 wt% Y_2_O_3_ 8.5–9.5 wt% HFO_2_ ≤ 0.5 wt% Al_2_O_3_ < 0.5 wt% other oxides < 0.1 wt%.	Amann Girrbach AG, Austria
CLEARFIL™ DC CORE PLUS	Dual‐cured composite core build‐up material	Resin; Bis‐GMA, TEGDMA. Filler: Silanated glass, silicaFiller loading: (74 wt%) 52 vol%. The particle size of 0.04–23 μm. Camphorquinone, Benzoylperoxide.	Kuraray, Japan
Clearfill SE	Two‐step, self‐etch adhesive	Primer: MDP, HEMA, dimethacrylate monomer, water, catalystBond: MDP, HEMA, dimethacrylate monomer, microfiller, catalyst.	Kuraray America, Houston, TX
Cojet™ sand	Silica‐coated Al_2_O_3_ particles	Aluminum oxide >95% Synthetic amorphous silica, fumed, Crystalline free <5%	Cojet™ Prep; 3 M™, Germany
Zircos‐E®	Etching solution	Hydrofluoric acid (HF), hydrochloric acid (HCl), sulfuric acid (H2SO4), nitric acid (HNO3), and phosphoric acid (H3PO4).0	(M & C Dental, Seoul, Korea).
RelyX™	Self‐adhesive resin cement powder	Glass powder, initiator, silica, substituted pyrimidine, calcium hydroxide, peroxy compound, pigment.	3 M™, Seefeld, Germany.
	Self‐adhesive resin cement liquid	Methacrylated phosphoric ester, dimethacrylate, acetate, stabilizer, initiator.	

**Figure 1:**
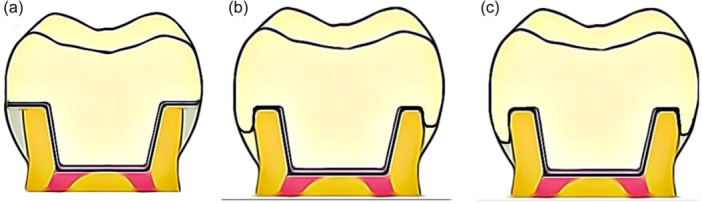
The preparation designs of mandibular molar teeth from left to right: (a) Endocrown with butt joint occlusal margin; no ferrule design. (b) Design with 1 mm ferrule. (c) Design with 2 mm ferrule.

The teeth were randomly allocated into three major groups based on the method of coronal preparation. Group N was the control group and received no additional preparation, with the endocrown having a butt joint design and a flat occlusal table. Groups F and F1 underwent 1‐ and 2‐mm circumferential ferrule preparations, respectively, apical to the endocrown occlusal surface. All‐access cavities were prepared to the same dimensions (4 ± 0.2 mm depth, 4 ± 0.2 mm buccolingually, and 6 ± 0.2 mm mesiodistally) with an 8°−10° inlay wall divergence and rounded internal angles. The teeth preparations were performed using a parallelometer to ensure standardized preparation parameters, and path of insertion and removal for all specimens (Figure 1).

**Figure 2:**
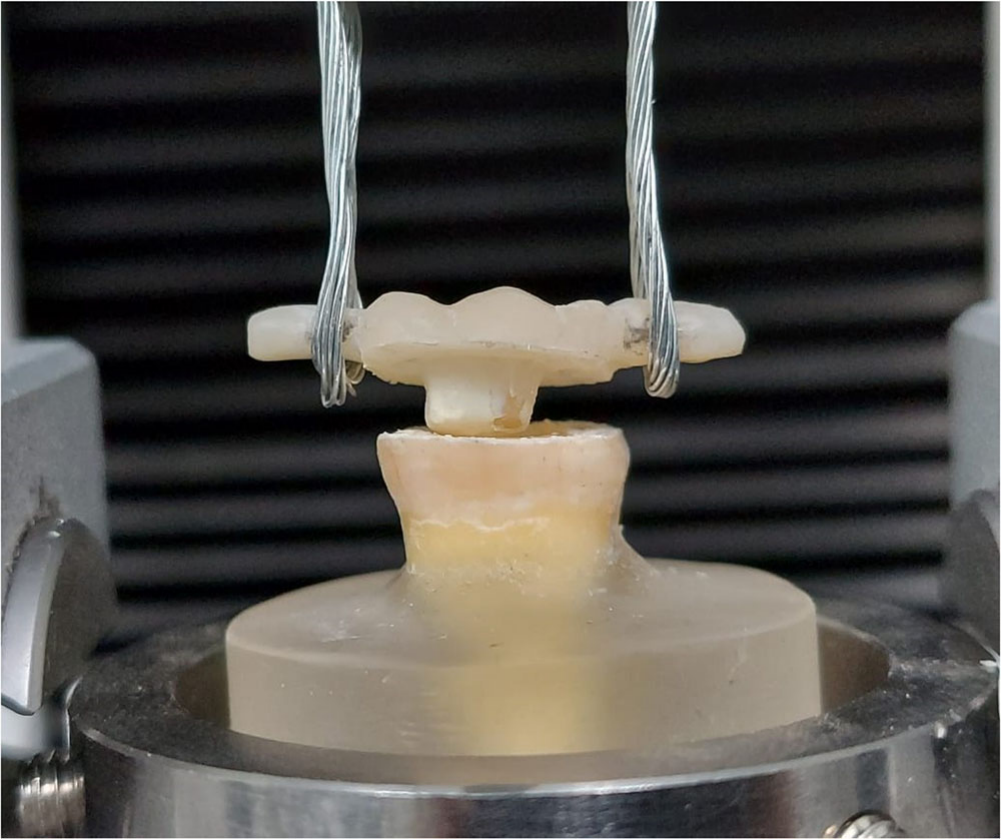
Showing the debonding mechanism of endocrown, the mesial and distal extended bars are hung to the pulling loops.

The prepared teeth were scanned by an intraoral scanner (TRIOS 3; 3Shape A/S, Germany). The endocrowns were then designed using computer‐aided design (CAD) software (3Shape CAD Design program, 3Shape), with a cement spacing of 60 µm. Subsequently, the endocrowns were milled from monolithic zirconia ceramic (5YSZ; Zolid FX, Amann Girrbach AG) utilizing a CAD/CAM milling machine (Ceramill; Amann Girrbach AG) following the manufacturer's guidelines. The final designs included a small flat bar extension on the mesial and distal sides of the occlusal surface to accommodate the dislodgment apparatus wire loops (Figure 2).

### 2.2 Surface treatment

The endocrowns in each preparation design group (*n* = 30) were assigned into three subgroups (*n* = 10) based on the zirconia surface treatment applied as follows;

Subgroup A: The intaglio surfaces of the endocrowns underwent air‐porn particle abrasion for 15 s with 50 μm Al_2_O_3_ particles from a 10 mm distance and a pressure of 0.3 MPa.

Subgroup Co: Specimen surfaces were air‐born particles abraded using silica‐coated Al_2_O_3_ (CoJet™ Sand; 3 M™) with a particle size of 30 μm under 2.8‐bar pressure for 15 s from a distance of 10 mm using the CoJet™ device (CoJet™ Prep; 3 M™).

Subgroup Z: specimens were etched for 30 min at 30°C using Zircos‐E® Etching Solution (M & C Dental).

All zirconia endocrowns were cleaned in an ultrasonic bath and gently dried with oil‐free air. The prepared teeth were cleaned for 15 s using a rubber cap and fluoride‐free pumice and washed with water. The endocrowns were then cemented with self‐adhesive resin cement (RelyX™ Unicem, 3 M™). The restorations were held in a specially designed device under a 4.9 N axially directed load for 5 min. Excess cement was removed, and light curing was performed for 20 s on each side of the endocrown restorations. The cemented endocrowns were placed in distilled water and stored in an incubator at 37°C for 24 h, before undergoing thermocycling for 5000 cycles with 5–55°C temperature fluctuations, and a dwell time of 15 s.

### 2.3 Retention test

To test the retention of the endocrowns, a universal testing machine (Instron; Instron Corp) was utilized. The specimens were dislodged at a crosshead speed of 0.5 mm/min until failure, either through debonding or fracture of the specimens (Figure 2). The dislodgment force (N) was measured for each endocrown. The mode of failure was categorized into favorable and non‐favorable, the debonded specimens with no damage of the restoration and the tooth was considered a favorable failure, and any fracture or damage of the restoration or the tooth was considered unfavorable. For debonded surfaces, the failure modes were further divided based on the criteria specified in Table 2.

**Table 2:** Failure mode distribution pattern after endocrown removal.

Category	Description	Nature
1	Cement retained mainly (75% and more) on the prepared tooth	Adhesive
2	Cement retained on both endocrown and the prepared tooth (25%–75%)	Cohesive
3	Cement retained mainly (75% and more) on endocrown fitting surface	Adhesive
4	Fracture of the tooth root without endocrown separation	Cohesive

### 2.4 Statistical analysis

To assess the assumption of normal distribution of the data on retention, we employed the Shapiro‐Wilk and Levene's tests. Considering the lack of significance in Levene's test regarding equal variances, a two‐way analysis of variance (ANOVA) was employed to determine the overall statistical significance of variations among the research variables. For pairwise statistical comparisons, Tukey's post‐hoc test was applied. In all tests conducted, statistical significance was defined as *p* ≤ .05.

## 3 RESULTS

All test specimens survived the thermocyclic aging. The values were normally distributed based on the Shapiro‐Wilk and Levene's test (*p* > .05). The two‐way ANOVA test indicated no significant interaction between the two variables tested (*p* = .108). The results showed a significant impact of the tooth preparation design (*p* < .001) and the type of surface treatment (*p* < .001) on the removal force of the endocrown. For all endocrown preparation designs, the debonding resistance was significantly higher for specimens abraded with silica‐coated particles and for those treated with Zircos‐E® Etching compared to those subjected to airborne‐particle abrasion with alumina particles. Similarly, for all three surface treatment protocols, the debonding resistance was significantly higher for the 2 mm ferrule design compared to the 1 mm ferrule design, and both exhibited significantly higher debonding resistance than endocrowns without a ferrule (Table 3).

**Table 3:** Endocrowns debonding resistance force mean and SD in *N*.

Zirconia surface	Design1		Design 2		Design 3	
	Mean	SD	Mean	SD	Mean	SD
airborne‐particle abrasion	357.00_a_^A^	36.22	588.10_a_^B^	53.15	658.50_a_^C^	87.24
Silica coating	531.35_b_^A^	60.31	670.53_b_^B^	73.62	755.00_b_^B^	64.68
Zircos‐E® Etching	535.60_b_^A^	36.82	677.10_b_^B^	65.58	776.00_b_^C^	94.77

*Note*: Upper superscripted capital letters indicate statistical significance within the raw, and the lower superscripted small letters indicate statistical significance within the column. Design 1: Endocrown with butt joint, flat occlusal table. Design 2: Endocrown with 1 mm circumferential ferrule. Design 3: Endocrown with 2 mm circumferential ferrule. *p* ≤ .05.

**Figure 3:**
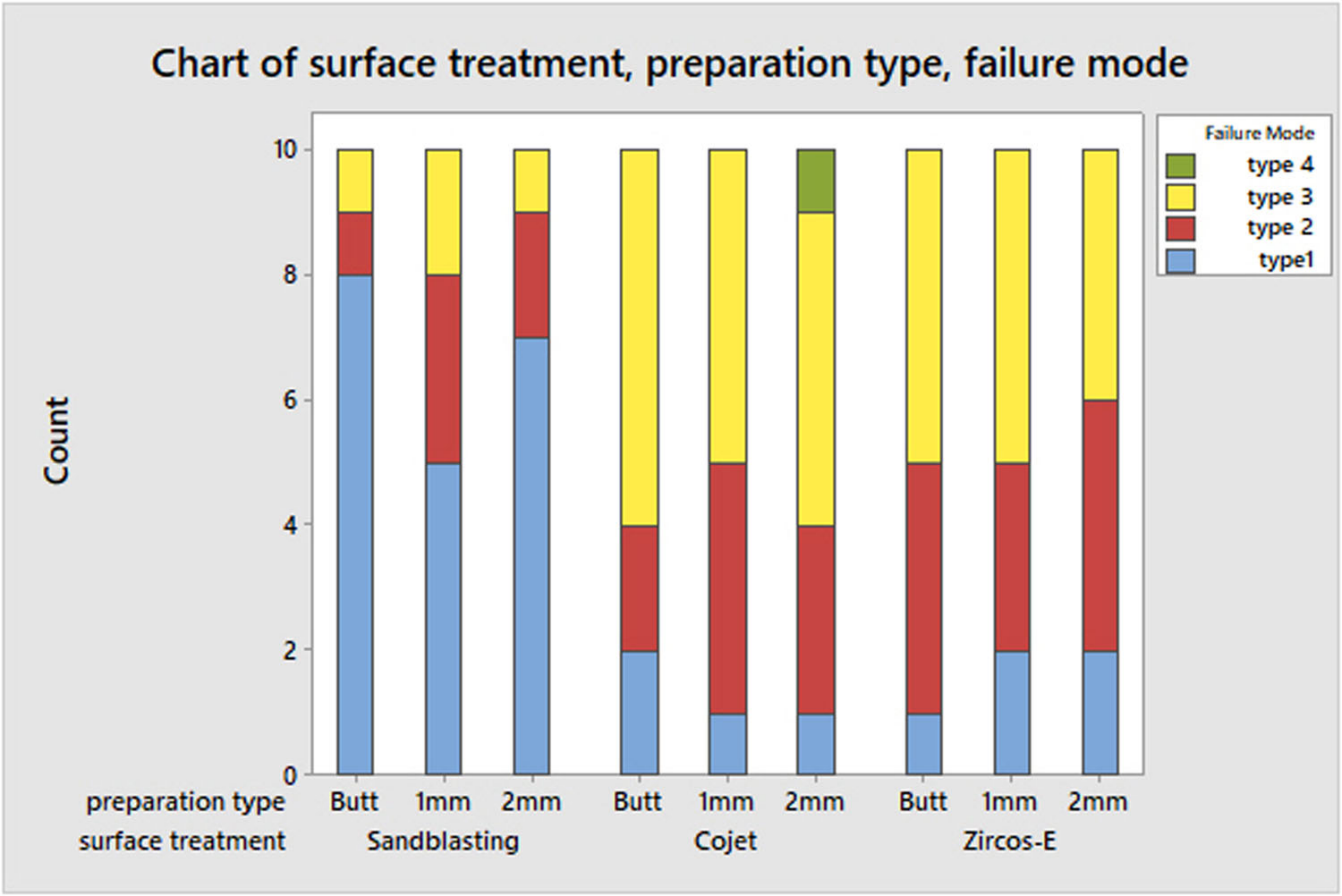
Chart showing the failure mode of debonded specimens.

Except for one endocrown with a 2 mm ferrule design and Cojet™ surface treatment, which fractured at the root without debonding, all other specimens showed favorable failure modes, either adhesive or cohesive within the cement layer. For zirconia endocrowns treated with air‐particle abrasion, the majority of failure modes were type 1, indicating adhesive failure between the cement and zirconia surface. Zirconia endocrowns treated with Cojet™ and Zircos‐E® Etching revealed higher cohesive failure in the cement layer and adhesive failure between the tooth substrate and cement (Table 2 and Figure 3).

## 4 DISCUSSION

The presented study aimed to evaluate the debonding resistance of 1 mm ferrule, 2 mm ferrule, and no ferrule endocrown designs on molar teeth, using three different zirconia surface pretreatments; alumina particle air‐particle abrasion; Cojet™ silica‐coating, and zircos‐E® etching. The debonding resistance of zirconia endocrowns was found to vary based on different designs and surface treatments, leading to the rejection of the first and second null hypotheses. However, the third null hypothesis was accepted as all specimens exhibited favorable failure modes, with no observed fractures or chipping except for one tooth that fractured in the root area without endocrown debonding.

Over the past decade, there has been a growing preference among dentists for the use of endocrowns as a contemporary approach to treating severely damaged ETT. This choice is driven by the advantages offered by endocrowns, particularly in molars, where they take advantage of the pulp chamber for mechanical interlocking and adhesion, eliminating the need for post‐and‐core crowns, which typically involve the removal of a significant amount of tooth structure (Carvalho et al., 2018; Soliman et al., 2021). There is conflicting literature regarding the association between posts and tooth root fractures (Carvalho et al., 2018). However, a clinical assessment has determined that endocrowns are the most suitable treatment option for patients with occlusal risk factors (Soliman et al., 2021), as they circumvent the potential for vertical root fracture in case of post‐core crowns (Lee et al., 2023). Consequently, endocrowns not only preserve tooth structure but also improve the clinical survival rate of severely damaged ETTs (Carvalho et al., 2018).

Given the challenges posed by relying on minimal tooth structure for retention in endocrowns, clinicians may opt for post‐and‐core crown options to enhance retention of larger restorations, considering that bonding to deep dentin is weaker than bonding to superficial dentin and bonding to pulpal dentin is weaker than bonding to coronal dentin (Deepa et al., 2014b; Kijsamanmith et al., 2002). Recent clinical studies have provided evidence to address these concerns, with a short‐term study showing a 100% survival rate for endocrowns compared to 66.7% for post‐and‐core crowns, (Morimoto et al., 2022) and a 5‐year study of 321 molar endocrowns demonstrating no failures according to USPHS criteria and a high patient satisfaction rate of 98%, attributed to the shorter treatment sessions and reduced time required compared to post‐and‐core crown methods. (Zou, Zhan, Xiang, & Li, 2022) However, it should be noted that in another study, an increased incidence of zirconia debonding was reported in comparison to lithium disilicate endocrowns, with three debonded zirconia endocrowns observed after 9, 10, and 13 months of clinical service out of 20 total restorations. (El‐Ma'aita, Abu‐Awwad, Hattar, & Devlin, 2022).

Debonding of monolithic ceramic endocrowns often occurs in severely destructed ETTs lacking axial walls (Belleflamme et al., 2017). To address this issue, researchers have explored increasing the depth of the pulpal inlay in endocrowns to enhance the bonding surface area. However, it has been found that this modification may not significantly impact the mechanical behavior of the restorations (Hayes et al., 2017). Moreover, increasing the pulpal depth of endocrowns can result in more noticeable discrepancies between fitting surfaces, (Gaintantzopoulou & El‐Damanhoury, 2016; Gan et al., 2023; Saker et al., 2024; Shin et al., 2017), and might negatively impact the fracture resistance of the endocrown restorations (Saker et al., 2024). The quality of the bond to dentin is influenced by various factors, including the location of the dentin substrate and the depth of preparation, indicating that coronal and superficial dentin provide better bonding substrates compared to deep and pulpal floor dentin (Deepa et al., 2014a). In the present study, a pulpal inlay depth of 3 mm was utilized. To ensure a uniform preparation of the inlay cavity and eliminate undercuts, a composite base material was applied, following recommendations from previous studies (Ahmed et al., 2022; Dietschi et al., 2008).

Based on the available literature, it appeared reasonable to expand the design of endocrowns to include the outer axial surface to avoid unnecessary inaccuracies and discrepancies between restorations and teeth‐fitting surfaces (Gan et al., 2023). This extension aims to achieve a more predictable, durable, and reliable bond with both the coronal and external superficial dentin (Deepa et al., 2014b; Kijsamanmith et al., 2002). The study was focused on molar crowns that had a height of 3 mm from the CEJ. Three different designs were adopted for the endocrowns: a butt joint without a ferrule extension and axial ferrule extensions of 1 and 2 mm, allowing the cervical and occlusal margins to be located on reliable outer enamel.

Zirconia, known for its chemically inert surface, requires pretreatment to achieve bonding. The literature presents conflicting results regarding the etching of zirconia ceramics, with some studies showing similar bond strength between etched and conventionally abraded surfaces, while recent research highlights improved bond strength using the Zircos‐E® etching solution (Sales et al., 2022; Sokolowski et al., 2023). Air‐particle abrasion is the commonly used technique for treating zirconia surfaces (Altan et al., 2019). Low jet pressure abrasion yields satisfactory results with luting resins containing MDP (Alghauli, Wille, et al., 2023; Hansen et al., 2020; Yang et al., 2018), while self‐adhesive resin cements require higher pressure, such as 0.25 MPa. In this study, a jet pressure of 0.3 MPa from a 10 mm distance for 15 s was employed (Yang et al., 2018).

For indirect partial coverage crowns without retentive means, the use of self‐adhesive resin cement may not be the most preferable option. Combining it with universal adhesives has been found to yield better outcomes (Alghauli, Alqutaibi, et al., 2023; Scholz et al., 2021). However, when it comes to indirect restorations with retentive inlays like endocrowns, favorable clinical and laboratory results have been observed (Alghauli, Alqutaibi, et al., 2023; Eltoukhy et al., 2021; Emam et al., 2023). Self‐adhesive cement has been recommended for the cementation of ceramic inlays and onlays. Clinical studies have shown that conditioning of dentin (Ghodsi et al., 2023) and etching of outer enamel (Peumans et al., 2013) are unnecessary for restorations with mechanical interlocking. This type of cement offers cost‐efficiency and simplified application, eliminating the need for complex protocols that can increase the risk of contamination and material application issues (Ghodsi et al., 2023).

The inclusion of a 2‐mm ferrule in the endocrown design demonstrated significantly higher debonding resistance compared to a 1‐mm ferrule design, and both ferrule designs exhibited greater debonding resistance than the design without a ferrule. This improvement is attributed to the increased surface area of the ferrule groups, enhancing adhesion by facilitating interlocking and friction during debonding. Previous studies have also reported the positive impact of a ferrule on the fracture strength of molar (Einhorn et al., 2019) and premolar teeth with endocrowns (Ahmed et al., 2022). A most recent laboratory study supported the findings of the current study. Nonetheless, the study evaluated only two endocrown designs on a molar, the conventional design and the design with a 2 mm ferrule (Buyukerkmen et al., 2022).

In terms of zirconia surface treatment, the resistance to debonding of Zircos‐E® etched zirconia specimens was nonstatistical and significantly different than Cojet™ silica‐coated air‐particle abraded specimens, while the two groups showed statistically significantly better resistance to debonding than alumina particle abraded zirconia specimens. These findings were supported by two recent studies published in 2022–2023, promoting Zircos‐E® etchant over air‐particle abrasion surface treatment (Sales et al., 2022; Sokolowski et al., 2023). Another study showed comparable bond strength of zirconia specimens treated with etchant or airborne‐particle abrasion (Sadid‐Zadeh et al., 2021). Airborne‐particle abrasion roughened the zirconia surface, generating microscopic irregularities, with deep valleys and peaks as seen under SEM. The etching of the zirconia surface, on the other hand, leaves a more homogenous surface topography (Sales et al., 2022; Sokolowski et al., 2023). The energy‐dispersive X‐ray analysis (EDAX) of alumina air‐particle abraded specimens showed traces of alumina which indicated the strong impaction of abrasive particles, some of them remain embedded in the zirconia surface. Moreover, the zirconia percentage on the air‐abraded surface was less than the etched surface, confirming the damaging effect of the air‐particle abrasion on the zirconia species (Sales et al., 2022), while the etched specimens revealed a reduced percentage of alumina (Sales et al., 2022; Sokolowski et al., 2023). However, Sokolowski et al. reported lower bond strength of etched specimens after aging, suggesting that airborne‐particle abrasion surface treatment is more durable in terms of bond strength (Sokolowski et al., 2023). In this study, the specimens underwent thermocycling for 5000 cycles, and the resistance to debonding was measured only after aging for all groups. The etched and silica‐coated zirconia specimens exhibited a higher force required to debond than alumina particle‐abraded specimens. Therefore, further investigations on the debonding resistance of endocrowns before and after aging or thermomechanical fatigue loading are recommended. Another laboratory study compared the retention force of zirconia endocrowns to leucite‐reinforced glass ceramic, ceramic‐infiltrated indirect composite, and zirconia‐reinforced lithium silicate (Emam et al., 2023). The study found comparable debonding resistance in all groups except for zirconia, which exhibited 50% of the debonding values of the other groups. This difference may be attributed to the lack of suitable surface pretreatment of the zirconia before adhesion, such as air‐particle abrasion or the use of zirconia etchants. These limitations should be taken into account.

The current study found that the lowest debonding resistance was observed in endocrowns without ferrule that underwent alumina particle abrasion surface treatment (357.00 ± 36.22). While this value was lower than the other groups, it still demonstrated a high load‐bearing capacity of 32.5–40 Kg, which is considered significant for a single restoration like the endocrown (Alghauli, Wille, et al., 2023; Chen & Fok, 2023). As a result, all designs and surface treatments evaluated in this study could perform well in clinical service. To optimize clinical outcomes, it is suggested to select either the 1 mm or 2 mm ferrule designs in combination with silica coating or Zirco‐E etching of the zirconia endocrown's intaglio surfaces, or to combine air‐particle abrasion with etching (Sokolowski et al., 2023). Additionally, to ensure long‐term survival and reliable bonding of the endocrown to the tooth substrate, the use of composite luting resins with 10‐MDP monomers or combining luting resin with an adhesive containing phosphate monomers is recommended (Buyukerkmen et al., 2022), and considering special treatment when bonding restorations to dentin such as immediate dentin sealing is beneficial for long term survival of both vital and endodontically treated teeth as in case of endocrowns (Alghauli et al., 2024).

The oral cavity simulation in this study was conducted in vitro using thermocycling in distilled water, without cyclic loading. It is important to acknowledge that various factors influence the bond strength and durability of endocrowns, including the type of adhesives and luting composite resins, adhesion strategies and conditions, tooth surface treatments, and materials. Therefore, further investigations are warranted. To assess the durability of the bonding between endocrowns and underlying tooth substrates, it is advisable to subject the specimens to rigorous testing conditions, particularly when considering the use of 10‐MDP containing luting resin and implementing fatigue or cyclic loading before debonding tests.

## 5 CONCLUSIONS

The following conclusions could be drawn:

1. The resistance of endocrowns to debonding can be improved by incorporating a ferrule with a height of 1 or 2 mm, thus increasing the surface area available for adhesion, compared to the non‐ferrule design.
2. Endocrowns treated with Zirco‐E etch and silica coating exhibited greater resistance to debonding compared to alumina particle abrasion. However, further laboratory research is necessary to simulate the long‐term survival of endocrown adhesion utilizing different luting composite resins, when subjected to severe fatigue loading conditions. Conducting a similar study on severely damaged molar teeth or other types of teeth, such as premolars, could offer further evidence.
3. The findings of this study are valuable for clinical decision‐making regarding the amount of the remaining tooth structure, the selection of appropriate design, and the suitable surface pretreatment for endocrown restorations.

## AUTHOR CONTRIBUTIONS

**Ahmed Yaseen Alqutaibi**: Conceptualization; methodology; investigation; results; writing the original draft; writing–review and editing; project administration. **Mohammed Ahmed Alghauli**: Conceptualization; methodology; investigation; results; writing original draft; writing, review and editing. **Karim Dewedar**: Results; writing the original draft; writing; review; supervision. **Karim Dewedar**: Methodology; investigation; results. **Mohammed H. AbdElaziz** and **Samah Mahmoud**: Investigation; results; project administration; supervision.

## CONFLICT OF INTEREST STATEMENT

The authors declare that they have no known competing financial interests or personal relationships that could have appeared to influence the work reported in this paper.

## ETHICS STATEMENT

The research protocol received approval from the Faculty of Dentistry ethics committee at Taibah University, with reference number # 21032 for the year 2022 and each patient voluntarily provided written consent after being fully informed.

## Data Availability

The datasets utilized in this investigation can be obtained from the corresponding author upon a reasonable request.
